# Genome-Wide Identification of Hsp70 Genes in the Large Yellow Croaker (*Larimichthys crocea*) and Their Regulated Expression Under Cold and Heat Stress

**DOI:** 10.3390/genes9120590

**Published:** 2018-11-29

**Authors:** Kaida Xu, Hanxiang Xu, Zhiqiang Han

**Affiliations:** 1 Key Laboratory of Sustainable Utilization of Technology Research for Fisheries Resources of Zhejiang Province, Scientific Observing and Experimental Station of Fishery Resources for Key Fishing Grounds, Ministry of Agriculture, Marine Fishery Research Institute of Zhejiang Province, Zhoushan 316002, China; xkd1981@dingtalk.com; 2 Fishery College, Zhejiang Ocean University, Zhoushan 316022, China; xuhanxiang@zjou.edu.cn

**Keywords:** heat-shock proteins, large yellow croaker, cold stress, heat stress, phylogenetic analysis

## Abstract

Heat shock proteins 70 (Hsp70) are required for key cellular processes and responses to environmental changes, however, there are an unknown number of *hsp70* gene family members in the large yellow croaker (*Larimichthys crocea*). In the present study, 17 *hsp70* genes were identified through the genome of the large yellow croaker. These genes are divided into seven evolutionarily distinct groups according to a phylogenetic tree. The orthologs of these *hsp70* genes were found in humans and zebrafish. The expression patterns of the *hsp70* gene family in the large yellow croaker under cold and heat stress were studied by examining transcriptome data. Six out of 17 genes were significantly unregulated or downregulated after cold or heat stress. There were two genes significantly upregulated and two genes downregulated in the liver after cold treatment, while after heat treatment, five genes were significantly upregulated, and no genes were significantly downregulated. Three expression patterns were detected: strictly heat-inducible *hsp70*, constitutively expressed and moderately heat-inducible *hsp70*, and constitutively expressed and less stress-dependent *hsp70* genes. All the findings will contribute to a better understanding of the biological function of *hsp70s* in defending against thermal challenges.

## 1. Introduction

Heat shock proteins (Hsps) are a large group of molecular chaperones. They are produced by cells in response to high temperature, hypoxia, infection, toxins, and a number of other types of stress. Some Hsps genes are named housekeeping proteins with constitutively expressed in non-stressed cells [1]. Heat shock proteins play crucial roles in restoring damaged proteins to their functional three-dimensional structure under external environmental stress [2]. Seven different major families, Hsp110, Hsp90, Hsp70, Hsp60, Hsp40, Hsp10, and small Hsps, are classified by their molecular weight [3]. Of these, the *hsp70* gene family is an important member of the Hsps superfamily, as it is widely distributed in all organisms from bacteria to mammals, except for some archaea [4,5,6].

Since they were first identified in the early 1960s [7], *hsp70* genes have been of great interest as they play critical roles in a series of biochemical processes, including the assembly of multimeric protein complexes, facilitating the intracellular folding of proteins, protein transportation across membranes, and the regulation of the heat-shock response [8]. They are expressed rapidly to protect organisms from environmental stress [9]. The heat shock proteins 70 gene family members provide a potent buffering system for adaption of cellular stress, either from extrinsic (pathogenic and environmental) or intrinsic stimuli (physiological, replicative, or oncogenic) [10].

The heat shock proteins 70 gene family members have different regulatory patterns at transcriptional level. The inducible members of *hsp70* genes (strictly inducible) exhibit a low basal level of expression and are only expressed under stress conditions, which typically lack introns. The stress includes, but is not limited to, heat stress [11]. The proteins of the heat-shock genes serve critical functions in protecting cellular proteins from damage under stressful conditions [12]. In contrast, the constitutively expressed Hsps (heat-shock cognates; *hsc70s*), which assist in the folding of newly translated proteins, are present in cells under all conditions. Heat-shock cognate 70 (*hsc70s*) typically contain several introns in the coding sequence [2].

The large yellow croaker, *Larimichthys crocea*, belongs to the family Sciaenidae, order Perciformes, and it is a temperate-water migratory fish [13]. It is mainly distributed from the southern Yellow Sea to the northern South China Sea [14]. As once one of the top four marine species in China, the catch production of large yellow croaker reached about 200,000 tons in the mid-1970s [15]. However, due to over-fishing spawn and overwinter stocks, fishery resources in terms of the large yellow croaker had suffered a severe decline since 1975 [15]. With the advance of artificial cultures, *L. crocea* has been gradually cultured in the coastal waters of the Zhejiang and Fujian provinces. The expansion of the artificial breeding scale and high density from the 2000s has led to the obvious decline of its immunity and caused lack of resistance to disease for survival in the aquatic environment [16]. High-density farming and temperature changes exacerbate the occurrence of diseases. The heat and cold stress have a serious impact on the survival rate of *L. crocea* [13]. The optimum water temperature for the growth of large yellow croaker is 18–25 °C. The large yellow croaker cannot tolerate high temperatures above 34 °C and low temperatures below 7 °C; the fish will reduce food intake when seawater temperatures are above 28 °C or below 14 °C [17,18].

Although many genetic studies have reported the expression pattern of some *hsp70* genes, the nomenclature for *hsp70* is exceptionally complex, generating a confusing array of data and naming conventions in databases and published literature. In some cases, it is impossible to know exactly which gene or protein in the family is being studied, since only the term *hsp70* is used without further clarification for the gene or protein [4].

Analyses of *hsp70* genes at the genome level to successfully identify new members have been carried out for a few species, such as humans, the channel catfish (*Ictalurus punctatus*), and the scallop (*Patinopecten yessoensis*) [1,4,10]. In humans, 17 identified genes were divided into seven evolutionarily distinct groups with distinguishable characteristic according to phylogenetic relationship [4]. In the channel catfish genome, 16 *hsp70* genes were detected and 10 out of 16 genes were significantly upregulated or downregulated after bacterial challenges [1]. However, in the large yellow croaker, only one member of the *hsp70* gene family has been identified and characterized [16]. There is still no genome-wide characterization of the *hsp70* gene family in the large yellow croaker to date. The large yellow croaker genomic resources and transcriptome sequences have been well developed in recent years [13,19]. These resources have made it feasible to conduct a systematic analysis of the genes of interest in the large yellow croaker genome.

A coherent, unifying informational framework encompassing all *hsp70* members that would facilitate an understanding of Hsps individually is lacking in teleost. Despite increasing demonstration of the role of *hsp70* genes in response to environmental stress in the large yellow croaker and other fish, some issues remain unclear. These include, for example, the constitution and evolutionary features of the *hsp70* gene family, the association between gene structure and function, and a systematic analysis of *hsp70* gene family involvement in environmental stress. In the present study, we conducted a genome-wide identification of a full set of *hsp70* genes and investigated their gene expression in the large yellow croaker under cold and heat stress. Seventeen *hsp70* genes were identified in the genome of the large yellow croaker, and the expression patterns in response to cold and heat were examined. Our observations highlight the molecular evolutionary properties and the response mechanism to temperature stress of *hsp70* genes in the large yellow croaker. Our findings will provide useful genetic resources to unravel the expression patterns and functions of *hsp70* genes in future studies and will contribute to understanding the evolutionary history of *hsp70* genes in teleosts.

## 2. Materials and Methods

The genome sequence, amino acid sequences, and transcriptome data of the large yellow croaker were downloaded from National Center for Biotechnology Information (NCBI) databases (accession number JRPU00000000.2) [20]. In order to identify the full set of *hsp70* genes, we used two strategies to search the large yellow croaker genome. First, BlastP (standard protein basic local alignment search tool (BLAST)) searches were performed against amino acid sequences of the large yellow croaker using *hsp70s* identified from humans and zebrafish as query sequences. Second, the hidden Markov model (HMM) profile of the Hsp70 domain (PF00012) was downloaded from the Pfam protein families database [21], and the HMM profiles of Hsp12a and Hsp12b (PTHR14187:SF46 and PTHR14187:SF3) were obtained from the PANTHER classification system [22]. These were exploited for the identification of *hsp70* genes from the yellow croaker genome using the software HMMER 3.0 [23]. The conserved domains of the candidate proteins were survey by online program SMART [24] and the National Center for Biotechnology Information (NCBI) conserved domain database [25]. Furthermore, the obtained full conserved domain sequences (CDS) of proteins from the large yellow croaker genome were used as queries to search against this species in RNA-seq datasets.

The protein sequences of *hsp70* genes identified from the large yellow croaker and other selected species were used to construct phylogenetic trees. The representative species for clustering and phylogenetic analysis were human (*Homo sapiens*), mouse (*Mus musculus*), platypus (*Ornithorhynchus anatinus*), chicken (*Gallus gallus*), Chinese softshell turtle (*Pelodiscus sinensis*), African clawed frog (*Xenopus laevis*), zebrafish (*Danio rerio*), medaka (*Oryzias latipes*), Nile tilapia (*Oreochromis niloticus*), torafugu (*Takifugu rubripes*), stickleback (*Gasterosteus aculeatus*), and anole lizard (*Anolis carolinensis*). The *hsp70* sequences from these species were retrieved from the NCBI (http://www.ncbi.nlm.nih.gov), Ensembl (http://asia.ensembl.org/index.html), and UniProt (http://www.uniprot.org) databases as listed by Song et al., 2016 [1] (Table S1). Multiple protein sequence alignments were conducted using the Multiple Sequence Comparison By Log-expectation (MUSCLE) method in the program MEGA 7.0 [26]. A maximum likelihood phylogenetic tree was constructed using MEGA 7.0 with a bootstrap test of 1000 replicates based on the LG + G model (amino acid replacement matrices incorporated the gamma distribution for modeling rate heterogeneity G) [27]. The LG + G model was selected as the best-fit model by the ProtTest program (version 3.4) [28].

The Multiple Expectation Maximization for Motif Elicitation software (MEME, version 4.8.1) was used to determine the conserved DNA sequence motifs in the *hsp70s* of large yellow croaker [29]. The parameters of MEME for detecting motif were the following: site distribution was set at 0 or 1 occurrence per sequence, the maximum number of motifs to be found was 15, and the motif width varied from 10 to 50. The diverse exon–intron organization of the *hsp70* genes was identified by comparing predicted coding sequences with their corresponding genomic sequences using the Gene Structure Display Server (version 2.0) [30].

### Expression Analysis of Heat Shock Proteins 70 Genes Under Thermal and Hypoxia Stress

To determine the expression profiles of *hsp70* genes after cold and heat treatment, the RNA-seq reads were retrieved from cold and heat challenge experiments in the large yellow croaker (SRR1964642-SRR1964649) [17]. A total of 160 individual large yellow croakers were divided in three groups: cold stress group (*n* = 60), heat stress group (*n* = 60), and control group (natural temperature, NT, *n* = 40). The temperatures for the three groups were 9 °C, 31 °C, and 22 °C, respectively. Liver tissues for transcriptome analysis were harvested from six fish 12 h after heat stress and cold stress.

After quality control, RNA sequencing reads were aligned independently with the large yellow croaker genome (GenBank accession no. JRPU00000000.1) [13] using TopHat (v2.0.3) software [31]. The mapped reads were then assembled into transcripts by Trinity (version 2.5.1), guided by a reference annotation of the large yellow croaker genome. Differential gene expressions were analyzed by RNA-seq by expectation-maximization (RSEM). The expression estimations of *hsp70* genes were normalized and represented in the form of RPKM (reads per kilobase per million mapped reads), and fold change (log_2_) values were calculated through the ratio of gene expression from the treated group compared to the control group. Genes with absolute log_2_ values > 2 and *t* test values (*p* < 0.05) after Bonferroni correction served as significantly differential expressed genes. Based on the log_2_ values, a heat map was generated by the Multiple Experiment Viewer 4.90 (MeV 4.90) [32].

Six significantly differential expressed genes were selected to validate RNA sequencing data by quantitative real-time polymerase chain reaction (qPCR) with gene specific primers designed using Primer v5.0 software (Premier Biosoft International). Primers are listed in Supplement Table S2. The ideal dilution times of cDNA samples were designed by the standard curves. The cDNA samples of template for PCR were diluted 20-fold with nuclease-free water in the procedure of qPCR analysis, following the manufacturer’s instructions for the SYBR^®^ Premix Ex TaqTM (Tli RNaseH Plus, Dalian, China). Using the ABI PRISM 7300 real-time PCR system (Applied Biosystems, Carlsbad, CA, USA) a reaction system of 25 µL was amplified. In order to increase the veracity of the result, three parallel experiments of every cDNA template were conducted The data were analyzed with ABI7300 SDS software (version 1.8, Applied Biosystems). The relative expression levels of all target unigenes or contigs were calculated by the 2^−ΔΔCT^ analysis method to analyze the expression level of genes. We chose the β-actin gene as a reference gene for internal standardization.

## 3. Results

### 3.1. Genome-Wide Identification of the Heat Shock Proteins 70 Gene Family in the Large Yellow Croaker

Basic local alignment search tool (BLAST) and HMM searches were conducted to extensively search *hsp70* genes in the genome of the large yellow croaker using human and zebrafish protein sequences as queries. A total of 26 putative *hsp70* genes were initially identified. Based on the valuation of Pfam and SMART scans, we discarded nine candidate sequences without the *hsp70* domain. Eventually, we obtained a total of 17 members representing the unique *hsp70* gene family in the large yellow croaker and created robust nomenclature following the Zebrafish Nomenclature Guidelines (https://wiki.zfin.org/display/general/ZFIN+Zebrafish+Nomenclature+Conventions).

The full length of coding sequences among these genes were identified in both transcriptome and genome databases. Detailed information about each *hsp70* gene is shown in Table 1. The predicted molecular weights varied from 46.4 kDa (*hspa12a*) to 110.2 kDa (*hyou1*). The *hspa12a* gene had the shortest conserved domain with 399 amino acids, and the longest domain (694 amino acids) was found in the gene *hspa4l.1*.

### 3.2. Phylogenetic Relationships of the Heat Shock Proteins 70 Genes Among Species

**Figure 1:**
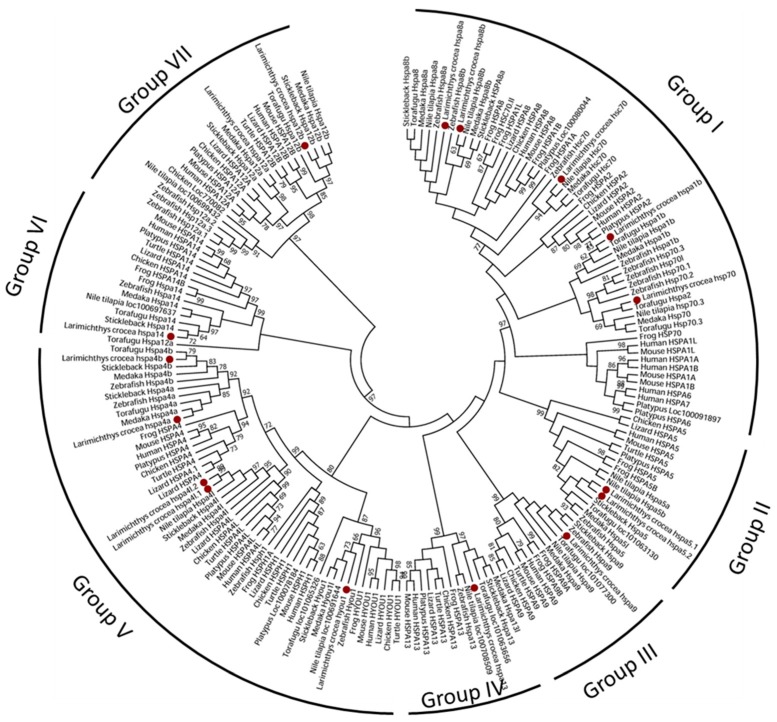
Phylogenetic tree of the heat shock protein 70 (Hsp70) family based on the maximum likelihood method. The bootstrap consensus tree inferred from 1000 replicates is taken.

Based on the amino acid sequences of Hsp70s, an unrooted phylogenetic tree for the *hsp70* family was generated to investigate the evolutionary relationships and elucidate classification in this gene family (Figure 1). Some gene duplications were revealed. Two copies of large yellow croaker *hsp70* genes were found highly orthologous to zebrafish *hspa5* and *hspa4l*, respectively. These were named *hspa5.1*, *hspa5.2*, *hspa4l.1*, and *hspa4l.2* after the zebrafish orthologies. Two related human *HSPA8A* genes were detected in the teleost fish genome, which were named *hspa8a* and *hspa8b* based on the cluster. The teleost-specific genes indicated that *hspa8a* and *hspa8b* were present after the divergence between teleost and Tetrapoda. The *hsp70s* gene names in the large yellow croaker were standardized following the zebrafish and human orthologs. The hsp70s from different species were more closely related to those in the same gene than to others in the same species. All members of the large yellow croaker *hsp70* gene family were well distributed into distinct groups and first clustered with corresponding genes of other fish species with strong bootstrap values (Figure 1).

The phylogenetic tree of *hsp70* family members from model species revealed seven major evolutionarily groups (Figure 1), with bootstrap support values over 87%. Group I, the largest group, included the *hsp70*, *hspa1a*, *hspa1b,* and *hspa8* genes. Group II, Group III and Group IV contained the *hspa5, hspa9*, and *hspa13* genes, respectively. Group V contained the closely related *hyou1*, *hspa4*, *hspa4L*, and *hspha1* genes. Group VI was composed of one gene, *hspa14.* Group VII was composed of the most diverged genes, *hspa12a* and *hspa12b*, which were quite dissimilar from the other *hsp70* genes.

The large yellow croaker has 12 homologous genes of *hsp70* that are also present in humans, with the exception of *hspa2*, *hspa6*, and *hspa7*. The *hsph1* gene was not found in any teleosts in the present study except in zebrafish. Most pairs of orthologs from the large yellow croaker and all other fish species were identified, indicating that the common ancestral genes of the *hsp70* family might have existed before the genetic divergence of fish species. In addition, two pairs of duplicated *hsp70* genes were found in the large yellow croaker, suggesting that *hsp70* genes underwent some duplication events after speciation. In Group I, three fish genes (*hspa8a*, *hspa8b*, and *hsc70*) showed a close relationship to the human *HSPA8*, showing possible paralogs (*hsc70* vs. *hspa8a* and *hspa8b*).

### 3.3. Gene Structure and Motif Analysis of the Heat Shock Proteins 70 in the Large Yellow Croaker

**Figure 2:**
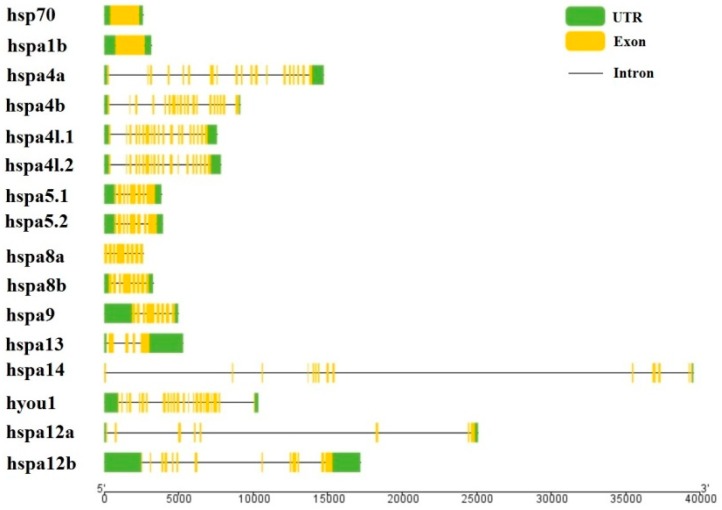
Exon-intron structure of large yellow croaker *hsp70* genes. Green boxes indicate untranslated 5′- and 3′-regions; yellow boxes indicate exons; black lines indicate introns.

We used the genome annotation file to analyze the gene structure of the large yellow croaker *hsp70* genes for better understanding the evolutionary conservation of this gene family. The exon-intron structure of each *hsp70* gene was showed in Figure 2. The intron–exon numbers vary greatly in these *hsp70* genes, indicating potential biological function diversities among those genes. However, the paralogous gene pairs derived from phylogenetic analysis shared a similar gene structure. However, the number of introns in these genes ranged from 0–13 (Figure 2). Based on the number of introns, genes could be divided into two patterns: pattern 1 with no introns and pattern 2 containing more than one intron. Only *hsp70* and *hspa1b* were assigned to pattern 1. Pattern 2 was common and contained 15 other genes.

**Figure 3:**
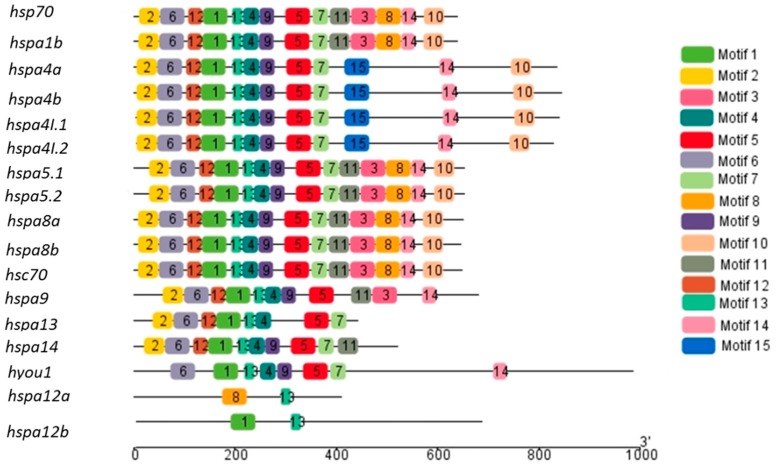
Distribution of conserved motifs in *hsp70* of the large yellow croaker. Each colored box represents a putative motif detected in the protein sequence.

To understand the functional diversification of the *hsp70* gene family, a conserved motif analysis was performed (Figure 3). We searched for 15 putative motifs in each gene as shown in Figure 3. In general, the Hsp70s from the same groups shared the similar motifs. The motifs were highly conserved within closely related *hsp70* members. Moreover, some *hsp70* from sister branches even had common motif compositions. Such phenomena were correlated with the gene structures and phylogenetic relationships. In the present study, the recently duplicated *hsp70* homologs exhibited similar motif arrangement architectures in their protein structures (Figure 3). Motifs 1, 2, 5, 6, 7, 11, and 13 were present in most *hsp70* genes. Only two motifs were detected in *hspa12a* and *hspa12b*.

### 3.4. Expression Regulation of the Heat Shock Proteins 70Gene Under Thermal Stress Treatment

**Figure 4:**
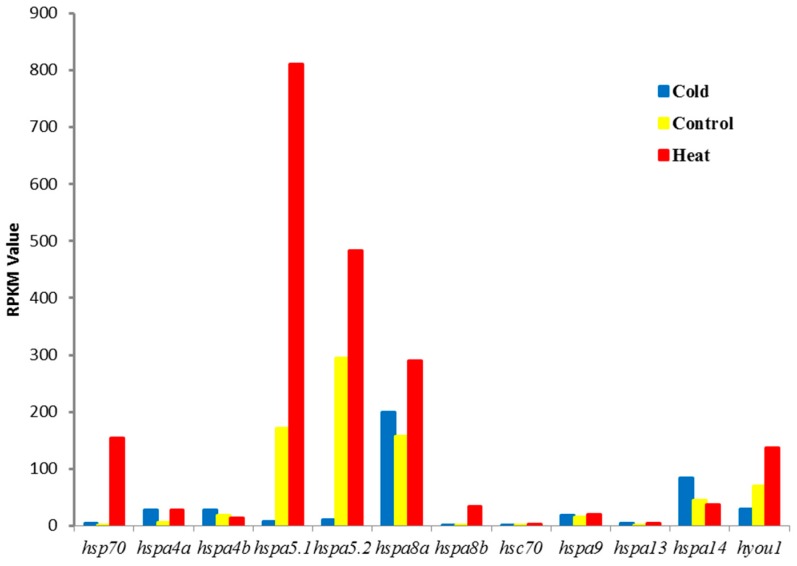
The *hsp70* expression of the large yellow croaker based on reads per kilobase per million mapped reads (RPKM) values.

**Figure 5:**
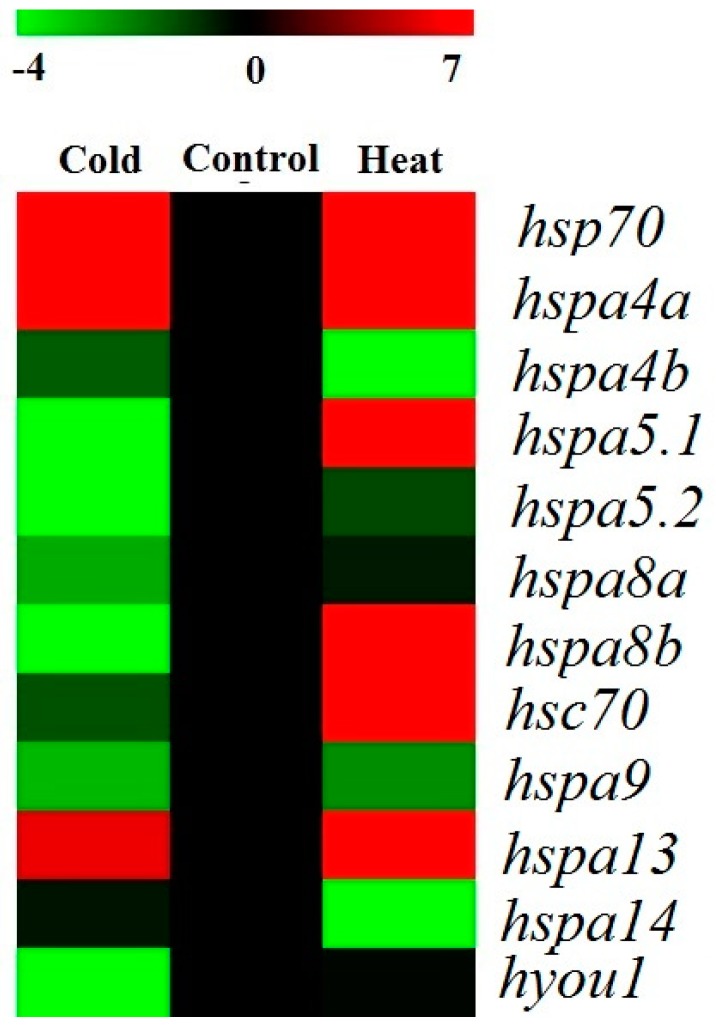
Heat map of *hsp70* expression under cold and heat stress treatments based on the fold change (log_2_) in RPKM values.

In order to assess the responses of the *hsp70* genes to thermal stress at the transcriptome level, RNA data from the livers of large yellow croakers after cold stress treatment, heat stress treatment, and normal temperature were analyzed. Based on these transcriptome data, the involvement of *hsp70* genes under cold and heat treatment was studied. Among the 17 *hsp70* genes, 12 members were expressed in liver tissue (Figure 4), which indicated a tissue expression pattern. As a result, six of the 17 *hsp70* genes were significantly differentially expressed by more than four-fold change (log_2_ fold change > 2) under cold treatment, including two upregulated genes (*hsp70, hspa4a*; log_2_ fold change: 2.03 to 2.17) and two downregulated genes (*hspa5.1* and *hspa5.2*; fold change: −4.49 to −4.85; Table 2, Figure 4 and Figure 5). Five significantly upregulated genes (log_2_ fold change: 2.01 to 7.32) were found under heat treatment, and no significantly downregulated genes were detected in this treatment (Table 2, Figure 4 and Figure 5). When comparing the cold and heat groups, five significantly upregulated genes (*hs70*, *hspa5.1*, *hspa5.2*, *hspa8b* and *hyou1*) were detected, while no significantly downregulated genes were found. The expression of six *hsp70* genes (*hspa4b*, *hspa8a*, *hspa9*, *hsapa13*, *hspa14*, and *hyou1*) showed little change in expression levels (Figure 4 and Figure 5) after thermal stress.

### 3.5. Validation of RNA-Seq Results by Quantitative Polymerase Chain Reaction

**Figure 6:**
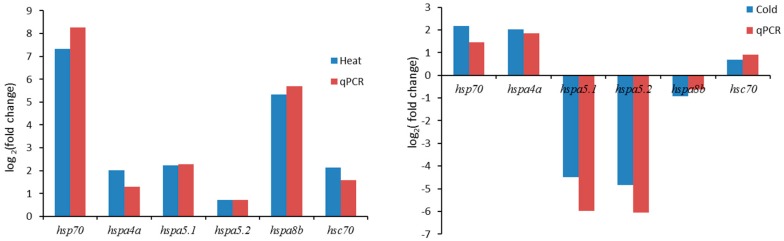
Validation of six significantly differential expressed genes by quantitative polymerase chain reaction (qPCR).

To validate the RNA-Seq results, six significant expressed genes were used for qPCR analysis (Figure 6). The results revealed that the expression trends of qPCR findings were consistent with the RNA-Seq results. Generally, the reliability and accuracy of the RNA-Seq analysis were confirmed by the qPCR results.

## 4. Discussion

Although regulated expression of one *hsp70* gene under thermal stress has been reported in the large yellow croaker [16], a systematic analysis of *hsp70* involvement in response to thermal stress had not been conducted in this species. Therefore, we performed an overall analysis of the *hsp70* gene family in the large yellow croaker, including an analysis of their gene structure, conserved motifs, phylogeny, and expression patterns under cold and heat stress.

A total of 17 *hsp70* genes were identified and annotated in the large yellow croaker. Compared to humans, most members of the *hsp70* gene family were found in the large yellow croaker, except *hspa2, hspa6, hspa7*, and *hsph1* found in humans Although exhaustive searches were conducted with genomic resources of the large yellow croaker, *hsph1*, *hspa6*, and *hspa7* were not found in the large yellow croaker genome. The *hsph1* gene was only found in zebrafish and was absent in other fish species, including the large yellow croaker, medaka, Nile tilapia, torafugu, and stickleback. The *hspa6* and *hspa7* genes were not found in any of the six fish species.

Early analyses based on sequences of typical Hsp70s in fish revealed two clusters, named “fish Hsp70-1” and “fish Hsp70-2” [9]. Our phylogenetic analysis of Hsp70s from humans, mice, chickens, zebrafish, the large yellow croaker, and other fish species can be sorted into seven distinct groups, which were supported by other sequence features, such as the exon–intron gene structure. The phylogenetic tree unveiled two major evolutionary events in fish Hsp70s: first, the duplication of *hspa4* into two separate genes (corresponding to *hspa4a* and *hspa4b*) in teleosts seems to have occurred after the divergence between teleosts and tetrapods (amphibians, reptiles, birds, and mammals). This was a newer duplication than the duplication of *hspa4* and *hspa4l*, which appeared to have occurred early in the evolution of the vertebrate lineage, before the appearance of tetrapods) [4]. Second, the close relation between the *hspa8a* and *hspa8b* genes in the large yellow croaker suggests that they duplicated after the divergence of the large yellow croaker and zebrafish, with the *hspa8a* gene in the zebrafish as a separate subcluster. Third, in the large yellow croaker, we found two recently diverged copies in *hspa4l* and *hspa5* (no sequence variation in CDS). The present results indicate that the teleost tended to have more duplications than mammals, likely as a consequence of whole genome duplication. In the channel catfish, three tandem repeats of *hspa8* were detected [1].

Six out of 17 *hsp70* genes were significantly upregulated or downregulated under thermal stress, indicating their involvement in thermal adaption. However, different expressed patterns were observed under cold and heat stress. There were two genes significantly upregulated and two genes downregulated in the liver after cold treatment, while after heat treatment, five genes were significantly upregulated and no genes were significantly downregulated. The different patterns may be attributed to the different influence of cold and heat on physiology. In general, cold stress usually reduces rates of enzymatic reactions, diffusion, and membrane transport (whereas heat stress would tend to accelerate these processes) [33]. Under cold stress, only two genes (*hspa5.1* and *hspa5.2*) were significantly downregulated. As an endoplasmic reticulum (ER) chaperone protein, the *hspa5* protein plays a key role in protein folding and quality control of protein synthesis in the ER lumen [34]. With decreasing temperature, the synthesis of proteins in the ER is restrained. Therefore, the expression of the *hspa5* genes participated in the correct folding of proteins and the degradation of misfolded proteins was limited by cold stress. The expression of *hspa5.1* was significantly upregulated under heat stress. The expression of the *hspa5* genes under cold and heat treatments was temperature induced. A similar result was reported in rainbow trout (*Oncorhynchus mykiss*). The *hspa5* mRNA expression of rainbow trout was significantly higher at 25 and 26 °C than that at 18 °C (*p* < 0.05) [35]. Based on the transcriptomic data for rainbow trout, *hspa5* was a significantly upregulated gene under heat stress [36]. Moreover, five genes (*hsp70*, *hspa4a*, *hspa5.1*, *hsc70*, and *hspa8b*) were significantly upregulated under heat stress. Under cold stress, *hspa4* and *hsp70* were also significantly upregulated. In the present study, there is a new finding for the *Hspa13* gene, which is induced by heat and cold stress in the large yellow croaker. However, the human *HSPA13* gene is induced by increases in cytoplasmic calcium but not by heat shock [37]. This finding indicated the different expression of *hspa13* in different species. Some studies have demonstrated the higher expression of the *hsp70* gene in zebrafish, rohu (*Labeo rohita*), and sea bass (*Dicentrarchus labrax*) after heat or cold stress [8,38,39]. The *hsp70* gene family can be distinguished by three expression patterns: strictly heat-inducible *hsp70*, constitutively expressed moderately heat-inducible *hsp70*, and constitutively expressed and less stress-dependent *hsp70* genes. Genes *hsp70* and *hspa8b* are the most inducible gene response to heat stress and belong to the group of strictly heat-inducible *hsp70* genes. In the large yellow croacker, *hspa4a*, *hsap5.1*, *hsap5.2*, *hsap8a*, and *hsc70* were constitutive and moderately induced by heat shock, which puts them in the group of moderately heat-inducible *hsp70* genes. The *hspa4b*, *hspa9*, *hspa14*, and *hyou1* genes, which belong to the group of constitutively expressed and less stress-dependent *hsp70* genes, were relatively highly expressed in the cold, control, and heat groups, but showed no significant expression between groups. Previous studies on the *hsp70* gene family mainly focused on strictly heat-inducible Hsp70s and cell-cycle-dependent heat-inducible *hsp70* genes. The previously identified two distinct groups of the Hsp70 gene family (fish Hsp70–1 and fish Hsp70–2) in reference [9] refer to the heat-inducible Hsp70 and cell-cycle-dependent heat-inducible *hsp70* genes.

Despite the lack of biological replicates in the transcriptome data [19], the expression results are still an indication. The pooling sample strategy in thermal treatments could have eliminated individual variation. The purpose of the present study was to provide a preliminary classification of all the members of the *hsp70* gene family in the genome of large yellow croaker. This is an essential step towards their unambiguous functional characterization. Our study will provide the basic foundation for further biological study to demonstrate the role of each large yellow croaker *hsp70* in thermal stress.

## Figures and Tables

**Table 1:** Summary of 17 *hsp70* genes identified in the large yellow croaker genome.

	Gene Name	Gene Accession Number	Protein Accession Number	CDS Length (bp)	Protein Length (aa)	Hsp70 Domain Location (aa)	Domain Feature	WT (kDa)
1	*hsp70*	XM_010755062.2	XP_010753364.1	1920	639	8–614	HSPA1-2-6-8-like_NBD	70.3
2	*hspa1b*	XM_010740692.2	XP_010738994.1	1917	638	7–613	HSPA1-2-6-8-like_NBD	70.3
3	*hspa4a*	XM_010729327.2	XP_010727629.1	2514	837	3–586	HSPA4_NBD	93.6
4	*hspa4b*	XM_010754752.2	XP_010753054.2	2535	844	3–598	HSPA4_NBD	94.6
5	*hspa4l.1*	XM_019277085.1	XP_019132630.1	2523	840	3–696	HSPA4_NBD	94.4
6	*hspa4l.2*	XM_019279519.1	XP_019135064.1	2493	830	3–557	HSPA4_NBD	93.5
7	*hspa5.1*	XM_010738795.2	XP_010737097.1	1965	654	28–634	HSPA5-like_NBD	72.2
8	*hspa5.2*	XM_019274093.1	XP_019129638.1	1965	654	28–634	HSPA5-like_NBD	72.2
9	*hspa8a*	XM_019279481.1	XP_019135026.1	1953	650	6–612	HSPA1-2-6-8-like_NBD	71.1
10	*hspa8b*	XM_019270840	XP_019126385.1	1941	646	6–612	HSPA1-2-6-8-like_NBD	70.9
11	*hsc70*	XM_010747566.2	XP_010745868.1	1950	649	6–612	HSPA1-2-6-8-like_NBD	71.07
12	*hspa9*	XM_010734059.2	XP_010732361.2	2043	680	55–653	HSPA9-like_NBD	73.4
13	*hspa12a*	XM_019257914.1	XP_019113459.1	1236	411	40–389	HSPA12A-like_NBD	46.4
14	*hspa12b*	XM_019255647.1	XP_019111192.1	2064	687	51–584	HSPA12B-like_NBD	76.5
15	*hspa13*	XM_010757137.2	XP_010755439.1	1329	442	33–431	HSPA13-like_NBD	48.3
16	*hspa14*	XM_019266323.1	XP_019121868.1	1566	521	18–521	HSPA14-like_NBD	56.4
17	*hyou1*	XM_019279502.1	XP_019135047.1	2961	986	28–690	HYOU1-like_NBD	110.2

CDS, conserved domain sequences; Hsp70, heat shock proteins 70; WT, molecular weight.

**Table 2:** Log_2_ fold change of large yellow croaker *hsp70* gene expression in liver under thermal stress. The significant genes (*p* value < 0.05, reads number >10, absolute log_2_ fold change >2) and their fold changes are denoted with *

Gene Name	*hsp70*	*hspa4a*	*hspa4b*	*hspa5.1*	*hspa5.2*	*hspa8a*	*hspa8b*	*hsc70*	*hspa9*	*hspa13*	*hspa14*	*hyou1*
Cold	2.17 *	2.03 *	0.64	−4.49 *	−4.85 *	0.33	−0.92	0.68	0.28	1.66	0.90	−1.27
Heat	7.32 *	2.01 *	−0.44	2.24 *	0.71	0.88	5.33 *	2.13 *	0.44	1.82	−0.25	0.95

## Supplementary Materials

The following are available online at http://www.mdpi.com/2073-4425/9/12/590/s1, Table S1: The species accession numbers of Hsp70 cited in the present study; Table S2: Primers used for quantitative real-time PCR (qPCR) validations.
