# Supplementary material for: Genome-Wide Identification of Hsp70 Genes in the Large Yellow Croaker (Larimichthys crocea) and Their Regulated Expression Under Cold and Heat Stress
Source: Genes (Basel). 2018 Nov 29;9(12):590. doi: 10.3390/genes9120590 (PMC6316144; doi:10.3390/genes9120590)
Supplement: Supplementary file 1 [file genes-09-00590-s001.zip › Table S1.pdf]

Table S1 The species accession numbers of Hsp70 cited in the present study

Associated Gene Name	NCBI Protein ID	Species Name	Latin name
Human_HSPA1A	NP_005336.3	human	*Homo sapiens*
Human_HSPA1B	NP_005337.2	human	*Homo sapiens*
Human_HSPA1L	NP_005518.3	human	*Homo sapiens*
Human_HSPA2	NP_068814.2	human	*Homo sapiens*
Human_HSPA4	NP_002145.3	human	*Homo sapiens*
Human_HSPA4L	NP_055093.2	human	*Homo sapiens*
Human_HSPA5	NP_005338.1	human	*Homo sapiens*
Human_HSPA6	NP_002146.2	human	*Homo sapiens*
Human_HSPA7	UniProtKB/Swiss-Prot: P48741.2	human	*Homo sapiens*
Human_HSPA8	NP_006588.1	human	*Homo sapiens*
Human_HSPA9	NP_004125.3	human	*Homo sapiens*
Human_HSPA12A	NP_079291.2	human	*Homo sapiens*
Human_HSPA12B	NP_443202.3	human	*Homo sapiens*
Human_HSPA13	NP_008879.3	human	*Homo sapiens*
Human_HSPA14	NP_057383.2	human	*Homo sapiens*
Human_HSPH1	NP_006635.2	human	*Homo sapiens*
Human_HYOU1	NP_001124463.1	human	*Homo sapiens*

Associated Gene Name	NCBI Protein ID	Species Name	Latin name
Mouse_HSPA1A	NP_034609.2	Mouse	*Mus musculus*
Mouse_HSPA1B	NP_034608.2	Mouse	*Mus musculus*
Mouse_HSPA1L	NP_038586.2	Mouse	*Mus musculus*
Mouse_HSPA2	NP_001002012.1	Mouse	*Mus musculus*
Mouse_HSPA4	NP_032326.3	Mouse	*Mus musculus*
Mouse_HSPA4L	NP_035150.3	Mouse	*Mus musculus*
Mouse_HSPA5	NP_001156906.1	Mouse	*Mus musculus*
Mouse_Hspa8	NP_112442.2	Mouse	*Mus musculus*
Mouse_HSPA9	NP_034611.2	Mouse	*Mus musculus*
Mouse_HSPA12A	NP_780408.1	Mouse	*Mus musculus*
Mouse_HSPA12B	NP_082582.1	Mouse	*Mus musculus*
Mouse_HSPA13	NP_084477.1	Mouse	*Mus musculus*
Mouse_HSPA14	NP_056580.2	Mouse	*Mus musculus*
Mouse_HSPH1	NP_038587.2	Mouse	*Mus musculus*
Mouse_HYOU1	NP_067370.3	Mouse	*Mus musculus*

Associated Gene Name	NCBI/ Ensemble Protein ID	Species Name	Latin name

Loc100091897	XP_001510204.2	Platypus	*Ornithorhynchus anatinus*
Loc100080044	XP_001510947.2	platypus	*Ornithorhynchus anatinus*
Platypus_HSPA2	ENSOANP00000015027	platypus	*Ornithorhynchus anatinus*
Platypus_HSPA4	ENSOANP00000011834	platypus	*Ornithorhynchus anatinus*
Platypus_HSPA4L	ENSOANP00000032051	platypus	*Ornithorhynchus anatinus*
Platypus_HSPA5	ENSOANP00000020985	platypus	*Ornithorhynchus anatinus*
Platypus_HSPA6	ENSOANP00000008582	platypus	*Ornithorhynchus anatinus*
Platypus_HSPA12A	ENSOANP00000022296	platypus	*Ornithorhynchus anatinus*
Platypus_HSPA13	ENSOANP00000004718	platypus	*Ornithorhynchus anatinus*
Platypus_HSPA14	ENSOANP00000025044	platypus	*Ornithorhynchus anatinus*
Loc100078184	XP_001509055.2	platypus	*Ornithorhynchus anatinus*

**Associated Gene Name**	**NCBI Protein ID**	**Species Name**	**Latin name**
Chicken_HSPA2	NP_001006686.1	chicken	*Gallus gallus*
Chicken_HSPA4	XP_003642142.1	chicken	*Gallus gallus*
Chicken_HSPA4L	NP_001012594.1	chicken	*Gallus gallus*
Chicken_HSPA5	NP_990822.1	chicken	*Gallus gallus*
Chicken_HSPA8	NP_990334.1	chicken	*Gallus gallus*
Chicken_HSPA9	NP_001006147.1	chicken	*Gallus gallus*
Chicken_HSPA12A	XP_421779.3	chicken	*Gallus gallus*
Chicken_Loc770082	XP_001233402.2	chicken	*Gallus gallus*
Chicken_HSPA13	NP_001025964.2	chicken	*Gallus gallus*
Chicken_HSPA14	XP_416996.3	chicken	*Gallus gallus*
Chicken_HSPH1	NP_001153170.1	chicken	*Gallus gallus*
Chicken_HYOU1	NP_001006588.1	chicken	*Gallus gallus*

**Associated Gene Name**	**Ensemble Protein ID**	**Species Name**	**Latin name**
Lizard_HSPA2	ENSACAP00000015494	Lizard	*Anolis carolinensis*
Lizard_HSPA4	ENSACAP00000013089	lizard	*Anolis carolinensis*
Lizard_HSPA4	ENSACAP00000023153	lizard	*Anolis carolinensis*
Lizard_HSPA4L	ENSACAP00000011642	lizard	*Anolis carolinensis*
Lizard_HSPA5	ENSACAP00000004078	lizard	*Anolis carolinensis*
Lizard_HSPA8	ENSACAP00000004798	lizard	*Anolis carolinensis*
Lizard_HSPA9	ENSACAP00000015698	lizard	*Anolis carolinensis*
Lizard_HSPA12A	ENSACAP00000009931	lizard	*Anolis carolinensis*
Lizard_HSPA12B	ENSACAP00000004138	lizard	*Anolis carolinensis*
Lizard_HSPA13	ENSACAP00000000965	lizard	*Anolis carolinensis*
Lizard_HSPA14	ENSACAP00000001088	lizard	*Anolis carolinensis*
Lizard_HYOU1	ENSACAP00000013983	lizard	*Anolis carolinensis*
Lizard_HSPH1	ENSACAP00000004913	lizard	*Anolis carolinensis*

Associated Gene Name	NCBI/ Ensemble Protein ID	Species Name	Latin name
HSPA4	enspsip00000014785	Chinese softshell turtle	*pelodiscus sinensis*
HSPA4L	enspsip00000010121	Chinese softshell turtle	*pelodiscus sinensis*
HSPA5	enspsip00000005890	Chinese softshell turtle	*pelodiscus sinensis*
HSPA9	enspsip00000014345	Chinese softshell turtle	*pelodiscus sinensis*
HSPA12B	enspsip00000004856	Chinese softshell turtle	*pelodiscus sinensis*
HSPA13	enspsip00000018220	Chinese softshell turtle	*pelodiscus sinensis*
HSPA14	enspsip00000018312	Chinese softshell turtle	*pelodiscus sinensis*
HYOU1	xp_006123893.1	Chinese softshell turtle	*pelodiscus sinensis*
HSPH1	enspsip00000009634	Chinese softshell turtle	*pelodiscus sinensis*

Associated Gene Name	NCBI Protein ID	Species Name	Latin name
HSPA1A	NP_001167480.1	African clawed frog	*Xenopus laevis*
HSPA1B	NP_001091238.1	African clawed frog	*Xenopus laevis*
HSPA1L	NP_001080068.1	African clawed frog	*Xenopus laevis*
HSP70	NP_001121147.1	African clawed frog	*Xenopus laevis*
HSPA2	NP_001086039.1	African clawed frog	*Xenopus laevis*
HSPA4	NP_001083317.1	African clawed frog	*Xenopus laevis*
HSPA5	NP_001081462.1	African clawed frog	*Xenopus laevis*
HSPA5B	NP_001165648.1	African clawed frog	*Xenopus laevis*
HSPA8	NP_001079632.1	African clawed frog	*Xenopus laevis*
HSC70.II	NP_001165656.1	AFRICAN CLAWED FROG	*XENOPUS LAEVIS*
HSPA9A	NP_001079627.1	African clawed frog	*Xenopus laevis*
HSPA9B	NP_001080166.1	African clawed frog	*Xenopus laevis*
HSPA13	NP_001017223.1	African clawed frog	*Xenopus laevis*
Hspa14	NP_001092168.1	African clawed frog	*Xenopus laevis*
HSPA14B	NP_001091353.1	African clawed frog	*Xenopus laevis*
HSPH1A	NP_001085637.1	African clawed frog	*Xenopus laevis*
HYOU1	UniProtKB/Swiss-Prot : Q566I3.2	African clawed frog	*Xenopus laevis*

Associated Gene Name	NCBI/ Ensemble Protein ID	Species Name	Latin name
Zebrafish_Hspa70.3	NP_571472.1	zebrafish	*Danio rerio*
Zebrafish_Hspa70.2	XP_003198158.1	zebrafish	*Danio rerio*
Zebrafish_Hspa70.1	**NP_001349288.1**	zebrafish	*Danio rerio*
Zebrafish_Hspa1b	NP_001093532.1	zebrafish	*Danio rerio*
Zebrafish_Hspa70l	NP_001107061.1	zebrafish	*Danio rerio*
Zebrafish_Hspa4a	NP_999881.1	zebrafish	*Danio rerio*
Zebrafish_Hspa4b	NP_956151.1	zebrafish	*Danio rerio*

Zebrafish_Hspa4l	XP_690505.2	zebrafish	*Danio rerio*
Zebrafish_Hspa5	NP_998223.1	zebrafish	*Danio rerio*
Zebrafish_Hspa8a	NP_001103873.1	zebrafish	*Danio rerio*
Zebrafish_Hspa8b[hsc70.2(LOC562935)]	NP_001186941.1	zebrafish	*Danio rerio*
Zebrafish_Hsc70	NP_956908.1	zebrafish	*Danio rerio*
Zebrafish_Hspa9	NP_958483.2	zebrafish	*Danio rerio*
Zebrafish_Hspa12a.1	NP_001038342.1	zebrafish	*Danio rerio*
Zebrafish_Hspa12a.2	XP_003198604.1	zebrafish	*Danio rerio*
Zebrafish_Hsp1a2a.3	NP_001038346.2	zebrafish	*Danio rerio*
Zebrafish_Hspa13	NP_001082948.1	zebrafish	*Danio rerio*
Zebrafish_Hspa14	NP_001038541.1	zebrafish	*Danio rerio*
Zebrafish_Hsph1	XP_001919957.1	zebrafish	*Danio rerio*
Zebrafish_Hyou1	NP_997868.1	zebrafish	*Danio rerio*

**Associated Gene Name**	**NCBI/Ensemble Protein ID**	**Species Name**	**Latin name**
Medaka_Hsp70.3(hspa)	XP_004071143.1	Medaka	*Oryzias latipes*
Medaka_Hsp70-5(hspa1b)	NP_001098384.1	medaka	*Oryzias latipes*
Medaka_Hsc70	NP_001098385.1	medaka	*Oryzias latipes*
Medaka_Hsc70.2(hspa8b)	XP_004075396.1	medaka	*Oryzias latipes*
Medaka_Hspa8a	UniProtKB/Swiss-Prot: Q9W6Y1.1	medaka	*Oryzias latipes*
Medaka_Hspa4a-201	ENSORLP00000001795	medaka	*Oryzias latipes*
Medaka_Hspa4b-201	ENSORLP00000007499	medaka	*Oryzias latipes*
Medaka_Hspa4l	XP_004082341.1	medaka	*Oryzias latipes*
Medaka_Hspa5l	XP_004074796.1	medaka	*Oryzias latipes*
Medaka_Hspa9-201	ENSORLP00000013340	medaka	*Oryzias latipes*
Medaka_Hspa12a-201	ENSORLP00000001447	medaka	*Oryzias latipes*
Medaka_Hspa12b-201	ENSORLP00000007349	medaka	*Oryzias latipes*
Medaka_Hspa13l	XP_004075919.1	medaka	*Oryzias latipes*
Medaka_Hspa14-201	ENSORLP00000015785	medaka	*Oryzias latipes*
Medaka_Hyou1l	XP_004084567.1	medaka	*Oryzias latipes*

**Associated Gene Name**	**NCBI Protein ID**	**Species Name**	**Latin name**
Nile tilapia_Hspa1lpartial(hsp70.3)	xp_003442504.1	nile tilapia	*oreochromis niloticus*
loc100704606 (hspa1b)	xp_003444871.1	nile tilapia	*oreochromis niloticus*

Nile tilapia_Hspa8a	xp_003448938.1	Nile tilapia	*oreochromis niloticus*
Hsc70	xp_003454400.1	nile tilapia	*oreochromis niloticus*
Nile tilapia_Hspa8b	xp_003455104.1	nile tilapia	*oreochromis niloticus*
Nile tilapia_Hspa4l	xp_003453147.1	nile tilapia	*oreochromis niloticus*
Nile tilapia_Hspa5a	xp_005470418.1	nile tilapia	*oreochromis niloticus*
Nile tilapia_Hspa5b	xp_003459659.1	nile tilapia	*oreochromis niloticus*
Nile tilapia_Hspa9	xp_003459471.1	nile tilapia	*oreochromis niloticus*
loc100699432	xp_003457416.1	nile tilapia	*oreochromis niloticus*
Nile tilapia_Hspa12b	xp_003452414.1	nile tilapia	*oreochromis niloticus*
loc100708509	xp_003441638.1	nile tilapia	*oreochromis niloticus*
loc100697637	xp_003455685.1	nile tilapia	*oreochromis niloticus*
loc100691644	xp_003448981.1	nile tilapia	*oreochromis niloticus*

**Associated Gene Name**	**NCBI/ Ensemble Protein ID**	**Species Name**	**Latin name**
Torafugu_Hsp70.3	XP_003964983.1	torafugu	*Takifugu rubripes*
Torafugu_Hspa1b	XP_003963154.1	torafugu	*Takifugu rubripes*
Torafugu_Hspa2-201	ENSTRUT00000005983	torafugu	*Takifugu rubripes*
Torafugu_Hspa8	XP_003977939.1	torafugu	*Takifugu rubripes*
Torafugu_Hsc70	XP_003966054.1	torafugu	*Takifugu rubripes*
Torafugu_loc101063130	XP_003965205.1	torafugu	*Takifugu rubripes*
Torafugu_loc101077300	XP_003977088.1	torafugu	*Takifugu rubripes*
Torafugu_loc101063656	XP_003968291.1	torafugu	*Takifugu rubripes*
Torafugu_Hspa4a	ENSTRUT00000021036	torafugu	*Takifugu rubripes*
Torafugu_Hspa4b	ENSTRUT00000016139	torafugu	*Takifugu rubripes*
Torafugu_Hspa12a	ENSTRUT00000009556	torafugu	*Takifugu rubripes*
Torafugu_Hspa12b	ENSTRUT00000006941	torafugu	*Takifugu rubripes*
Torafugu_Hspa14	ENSTRUT00000031936	torafugu	*Takifugu rubripes*
Torafugu_ loc101065326	XP_003977948.1	torafugu	*Takifugu rubripes*

**Associated Gene Name**	**Ensemble Protein ID**	**Species Name**	**Latin name**
Stickleback_Hspa4a	ENSGACP00000027410	Stickleback	*Gasterosteus aculeatus*
Stickleback_Hspa4b	ENSGACP00000024055	stickleback	*Gasterosteus aculeatus*
Stickleback_Hspa4l	ENSGACP00000010866	stickleback	*Gasterosteus aculeatus*
Stickleback_Hspa5	ENSGACP00000021969	stickleback	*Gasterosteus aculeatus*
Stickleback_HSPA8a	ENSGACP00000013930	stickleback	*Gasterosteus aculeatus*
Stickleback_Hspa8b	ENSGACP00000026579	stickleback	*Gasterosteus aculeatus*
Stickleback_Hspa9	ENSGACP00000025843	stickleback	*Gasterosteus aculeatus*
Stickleback_Hspa12a	ENSGACP00000019311	stickleback	*Gasterosteus aculeatus*
Stickleback_Hspa12b	ENSGACP00000026246	stickleback	*Gasterosteus aculeatus*
Stickleback_Hspa13	ENSGACP00000008388	stickleback	*Gasterosteus aculeatus*
Stickleback_Hspa14	ENSGACP00000025513	stickleback	*Gasterosteus aculeatus*

Stickleback_Hyou1	ENSGACP00000026575	stickleback	*Gasterosteus aculeatus*
